# Lipid Transfer Proteins and Membrane Contact Sites in Human Cancer

**DOI:** 10.3389/fcell.2019.00371

**Published:** 2020-01-23

**Authors:** Diego Peretti, SoHui Kim, Roberta Tufi, Sima Lev

**Affiliations:** ^1^UK Dementia Research Institute, Clinical Neurosciences Department, University of Cambridge, Cambridge, United Kingdom; ^2^Nakseongdae R&D Center, GPCR Therapeutics, Inc., Seoul, South Korea; ^3^MRC Mitochondrial Biology Unit, University of Cambridge, Cambridge, United Kingdom; ^4^Molecular Cell Biology Department, Weizmann Institute of Science, Rehovot, Israel

**Keywords:** LTPs, MCSs, cancer, calcium, lipids

## Abstract

Lipid-transfer proteins (LTPs) were initially discovered as cytosolic factors that facilitate lipid transport between membrane bilayers *in vitro*. Since then, many LTPs have been isolated from bacteria, plants, yeast, and mammals, and extensively studied in cell-free systems and intact cells. A major advance in the LTP field was associated with the discovery of intracellular membrane contact sites (MCSs), small cytosolic gaps between the endoplasmic reticulum (ER) and other cellular membranes, which accelerate lipid transfer by LTPs. As LTPs modulate the distribution of lipids within cellular membranes, and many lipid species function as second messengers in key signaling pathways that control cell survival, proliferation, and migration, LTPs have been implicated in cancer-associated signal transduction cascades. Increasing evidence suggests that LTPs play an important role in cancer progression and metastasis. This review describes how different LTPs as well as MCSs can contribute to cell transformation and malignant phenotype, and discusses how “aberrant” MCSs are associated with tumorigenesis in human.

## Introduction

Lipid-transfer proteins (LTPs) are highly conserved lipid carriers that bind monomeric lipids in a hydrophobic pocket, and transfer them between donor and acceptor membranes through an aqueous phase (Zilversmit, 1983; Holthuis and Levine, 2005). Based on their lipid binding specificity, LTPs can be divided into several subgroups including: (1) sphingolipid-, (2) sterol-, and (3) phospholipid-transfer proteins (Lev, 2010). A close proximity between the donor and the acceptor membranes, as occurs at MCSs, reduces the diffusion distance of LTPs and accelerates intermembrane lipid transport. Although LTPs were discovered in the late 1970s (Wirtz, 1974; Wirtz et al., 1980) and MCSs already observed by electron microscopy in the 1950s (Porter, 1953), their physiological functions and regulatory properties have only been emerged in the last few years (Levine, 2004; Selitrennik and Lev, 2016).

Numerous studies on LTPs and MCSs from the last five years highlighted their important roles in regulating intracellular lipid distribution and signaling, and demonstrated the diversity of MCSs, their dynamics, tethering mechanisms, and various physiological functions (Saheki and De Camilli, 2017). These studies suggest that LTPs and MCSs are involved in central cellular processes, including cell growth and migration, cellular metabolism, and proteostasis (Sassano et al., 2017). Abnormal regulation of these processes is frequently associated with tumorigenesis, implying that LTPs and MCSs can contribute to tumor development and metastasis.

Indeed, increasing evidence suggests that LTPs can modulate local lipid composition of membranes, and thus, influence their biophysical properties (fluidity, curvature) as well as the content of lipid second messengers (van Meer, 1993; Levine, 2007; Van Meer et al., 2008). Of the various lipid second messengers, phosphoinositides, and in particular, phosphatidylinositol-3,4,5-trisphosphate (PIP_3_) and its precursor phosphatidylinositol-4,5-bisphosphate (PIP_2_) are tightly associated with human cancer (Toker, 2002; Brown and Toker, 2015). Other signaling lipids such as sphingolipids and fatty acids also play a role in cancer progression and metastasis (Luo et al., 2018), and further information on the function of lipids and lipid metabolism in cancer can be found elsewhere (Murai, 2015; Kim et al., 2016; Long et al., 2018). In this review, we discuss the role of several LTPs, including phosphatidylinositol (PI)-transfer proteins (PITPs) and steroidogenic acute regulatory protein (StAR)-related lipid transfer (START family) (Soccio and Breslow, 2003; Alpy and Tomasetto, 2005) in human cancer, and further describe the heterogeneity of MCSs, their function in lipid transport and calcium signaling, and their implication in cancer biology. Additional information related to LTPs and MCSs had been previously described in many excellent reviews and are not covered here (Cohen et al., 2018; Prinz et al., 2019; Scorrano et al., 2019; Wong et al., 2019).

## Phosphoinositides and Cancer

All phosphoinositides are derivatives of PI, a phospholipid that is synthesized in the ER and is composed of a hydrophobic diacylglycerol (DAG) coupled to inositol 1-monophosphate ring (Lev, 2012). Phosphorylation of the inositol ring at its 3, 4, and 5 hydroxyl groups, either at single site or in combination, results in the seven different phosphorylation states of membrane phosphoinositides, including PI3P, PI4P, PI5P, PI(3,4)P_2_, PI(3,5)P_2_, PI(4,5)P_2_, and PI(3,4,5)P_3_. These phosphoinositides are distinctly distributed between intracellular organelles and play different cellular functions (Balla, 2013). PI(3)P and PI(3,5)P_2_ are considered as endolysosomal species, PI4P is enriched in the *trans*-Golgi network (TGN) and PI5P within the nuclei, whereas PI(4,5)P_2_, PI(3,4)P_2_, and PI(3,4,5)P_3_ are mainly found at the plasma membrane (PM) (De Craene et al., 2017). The production and maintenance of these different phosphoinositides is mediated by a network of interconverting enzymes including phosphoinositide-specific kinases and phosphatases.

Although phosphoinositides are minor phospholipids of the PM, PI(4,5)P_2_, which plays a central role in cellular signaling, is considered to be the most abundant. It undergoes rapid hydrolysis by phospholipase C (PLC) in response to multiple external stimuli to generate DAG and inositol-1,4,5-trisphosphate (IP_3_) second messengers. In addition, it binds to proteins that regulate actin polymerization, cell adhesion and cell-cell contact, and consequently affects cancer cell motility (Bunney and Katan, 2010). Most importantly, PI(4,5)P_2_ is phosphorylated by PI3K (phosphatidylinositol 3-kinase) to generate PI(3,4,5)P_3_, an important phosphoinositide that regulates cell survival, proliferation and growth. PI(3,4,5)P_3_ can be dephosphorylated by the 3′-phosphatase PTEN to terminate PI3K signaling. Notably, activating mutations in the catalytic domain of PI3K, i.e., PIK3CA, and loss-of-function mutations in PTEN are among the most common genetic alterations found in human cancer, demonstrating the central role of this phosphoinositide in cancer biology (Engelman, 2009). In addition, AKT which is activated by PI(3,4,5)P_3_, is amplified, overexpressed or hyperactivated in multiple human cancers (Altomare and Testa, 2005). Given the central role of PI(3,4,5)P_3_ in human cancer, it is not surprising that inhibition of PI(3,4,5)P_3_ production and/or its downstream effectors utilizing kinase inhibitors to PI3K, AKT, or mTOR (mechanistic target of rapamycin) have been utilized as promising strategies for cancer therapy (Engelman, 2009).

Recent studies, however, suggested that several phosphoinositide-transfer proteins also regulate PI(3,4,5)P_3_ levels and are implicated in cancer progression and metastasis. We discuss a few examples including, PITPα and β, Nir2, PITPNC1, and TIPE3.

### PITPs

**FIGURE 1:**
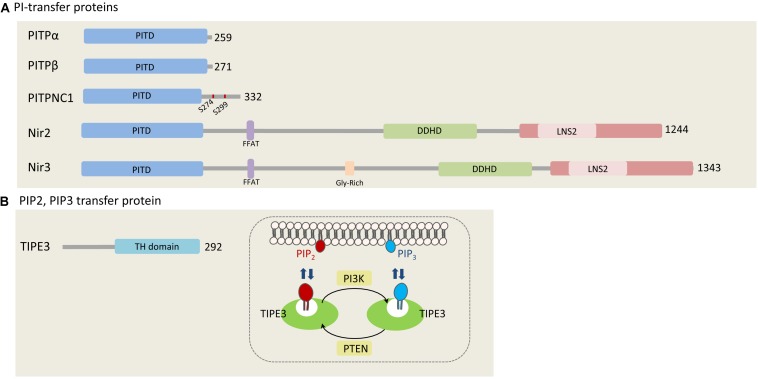
Phosphatidylinositols transfer proteins. **(A)** PI-transfer proteins. The five human PI-transfer proteins can be divided into small proteins consisting of a single PI-transfer domain (PITD) including PITPα/β and PITPNC1, and the multi-domains containing proteins Nir2 and Nir3. Shown are the PITD, the FFAT motif, DDHD, and the C-terminal LNS2 (Lipin/Nde1/Smp2) domain. Glycine rich region is found only in Nir3 (Lev, 2004). PITPNC1 phosphorylation sites (S274 and S299), which bind 14-3-3, are represented as red dots on PITPNC1 protein (Halberg et al., 2016). **(B)** TIPE3, a PIP_2_, and PIP_3_ transfer protein. TIPE is the only protein that is known to transfer phosphoinositides. It preferentially binds PIP_2_ and PIP_3_, and contributes to increase their levels at the PM by mediating efficient supply of PIP_2_ and presenting it to PI3K to produce PIP_3_ (Fayngerts et al., 2014). The numbers at the right side of each protein indicate the length of each protein in amino acids.

In humans, there are five PITPs that can be classified into two major groups: small PITPs, which include PITPα, PITPβ, and PITPNC1, and large multi-domains proteins including Nir2 and Nir3 (Figure 1A; Lev, 2004). The PI-transfer domain is highly conserved in all human PITPs and can transfer PI and phosphatidylcholine (PC), whereas few PITPs can also transfer phosphatidic acid (PA) and sphingomyelin (SM) (Li et al., 2002; Yadav et al., 2015).

The involvement of PITPα and β in phosphoinositides production, turnover and signaling has been demonstrated by many studies employing reconstituted systems, cell-free assays and intact cells. Collectively, these studies showed that PITPα and β can enhance PI(4,5)P_2_ and PI(3,4,5)P_3_ production (Cockcroft and Garner, 2013). In addition, it was shown that overexpression of PITPα in mouse fibroblasts markedly enhanced cell proliferation (Schenning et al., 2004), and that depletion of Nir2 by shRNA substantially reduced PI(4,5)P_2_ levels at the plasma membrane and consequently PI(3,4,5)P_3_ production in response to growth factor stimulation (Chang et al., 2013; Kim et al., 2013; Chang and Liou, 2015). Low levels of these phosphoinositide second messengers were accompanied by reduced AKT and ERK1/2 phosphorylation, and as a result, inhibition of cell migration and invasion (Keinan et al., 2014). Nir2 depletion markedly attenuated the migration and invasion of mammary epithelial cells and human breast carcinoma and induced mesenchymal-to-epithelial transition (MET) of highly metastatic breast cancer cells. Consistent with these findings, we showed that Nir2 level was upregulated during EMT, and its depletion in breast cancer blocked lung metastasis in animal models (Keinan et al., 2014). We also observed high correlation between Nir2 expression and tumor grade as well as poor disease outcome of breast cancer patients.

PITPNC1 is also implicated in cancer metastasis, but in contrast to PITPα and β, has a unique C-terminal extension with two serine phosphorylation sites, which provide docking sites for 14-3-3 protein (Garner et al., 2011). It was proposed that 14-3-3 binding protects PITPNC1 from degradation and inhibits its lipid transfer activity (Cockcroft and Garner, 2012). While further studies should explore this hypothesis, currently there is strong evidence that PITPNC1 is associated with different human cancers. It is highly expressed in several cancers, and its overexpression significantly correlates with metastatic progression of breast, melanoma, and colon cancers. PITPNC1 was identified as a target gene of miR-126, a metastasis suppressor microRNA (Png et al., 2012). It is amplified in a large fraction of human breast cancers, and its depletion by shRNA markedly attenuated metastasis in animal models (Halberg et al., 2016). Mechanistic studies suggest that PITPNC1 binds PI4P and enhances the secretion of pro-invasive and pro-angiogenic mediators, through recruitment of RAB1B (Ras-related protein Rab-1B) and the PI4P-binding protein GOLPH3 (Golgi phosphoprotein 3) to the TGN (Halberg et al., 2016). Interestingly, PITPNC1 was also found to bind and transfer PA but not PC (Garner et al., 2012), implying that it has unique lipid binding and/or transfer capabilities.

### TIPE3

TIPE3 belongs to the TNFAIP8 (tumor necrosis factor-alpha-induced protein 8, or TIPE) family of proteins which are implicated in tumorigenesis and inflammation (Moniz and Vanhaesebroeck, 2014). It contains a C-terminal TIPE2 Homology (TH) domain, consisting of a large hydrophobic cavity that accommodates phospholipid molecules (Fayngerts et al., 2014). Similarly to the other TNFAIP8 members (TIPE1, TIPE2, and TNFAIP8), TIPE3 can bind a number of phosphoinositides, including PI(4,5)P_2_, PI(3,5)P_2_, PI(3,4)P_2_, and PI(3,4,5)P_3_. It preferentially captures and transfers PI(4,5)P_2_ and PI(3,4,5)P_3_ and increases their levels at the PM, thereby promoting AKT and ERK pathways activation (Fayngerts et al., 2014). It was proposed that TIPE3 functions as a lipid-presenting protein and enhances PI(3,4,5)P_3_ production by PI3K (Figure 1B).

TIPE3 is highly expressed in several human cancers including lung, cervical, colon, esophageal and breast. Its overexpression enhances cell growth, migration and invasion *in vitro* and tumor growth in animal models, whereas its knockdown has opposite effects (Fayngerts et al., 2014; García-Tuñón et al., 2017). These observations suggest that TIPE3, and possibly its other family members, are a new class of phosphoinositide transfer proteins, which regulate tumor growth and progression.

## Start Proteins and Their Involvement in Human Cancer

**FIGURE 2:**
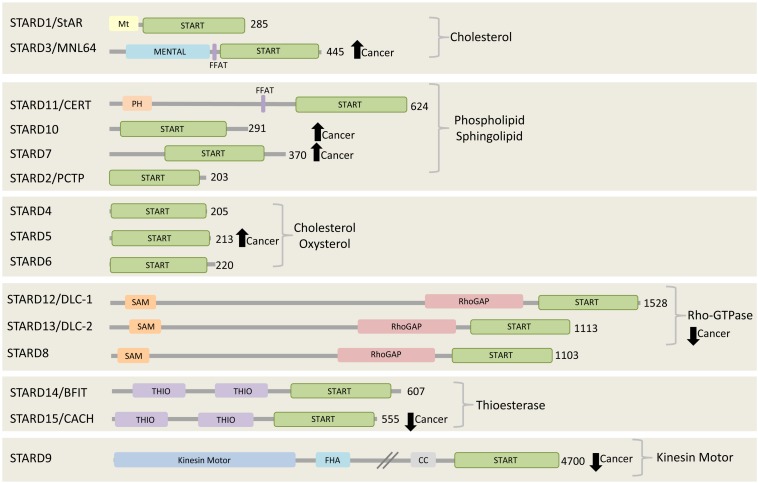
The START proteins. Fifteen START proteins in human are grouped into six subfamilies. Three groups share the indicated lipid binding/transfer specificity of START domain, while the other three groups share the indicated functional domains. All members have their START domain at the C-terminal region. Among 15 START proteins, two of them, STARD3 and CERT, contain FFAT motif. STARD3, STARD10, STARD7, and STARD5 are found to be highly expressed and connected to poor prognosis in various cancers including breast cancer, gestational trophoblastic tumor (Clark, 2012). On the other hand, the expression of all members of Rho-GTPase subgroup, STARD8/12/13, STARD9, and STARD15 are reported to be decreased in cancer (Clark, 2012). The number at the right side of each C-terminal represents the length of each protein in amino acids.

In mammals, there are fifteen proteins containing the START (StAR-related lipid-transfer) domain, which can be grouped into six subfamilies according to sequence similarities and lipid binding specificities. The STARD1/D3 subfamily has specificity for cholesterol, STARD4/D5/D6 subfamily for cholesterol or oxysterol, and STARD2(PCTP)/D7/D10/D11 subfamily for phospholipids or sphingolipids (Figure 2). The lipid-binding specificity of the other three subgroups is unknown, but they share other functional domains. STARD8/12/13 subfamily shares a putative Rho-GTPase domain, STARD14/15 subfamily has thioesterase activity, and STARD9 has a kinesin motor function (Alpy and Tomasetto, 2005). Interestingly, the START domain is always located at the C-terminal of the START proteins, possibly to facilitate lipid binding, transfer and release. Few START proteins contain membrane targeting motifs that mediate their interaction with different organelles. STARD1, for example, has a mitochondrial targeting motif and STARD3 has a MENTAL (MLN64 NH(2)-terminal) domain for late endosome (LE) targeting, while STARD11/CERT (ceramide transfer protein) contains a PH (pleckstrin homology) domain for PI4P binding at the Golgi complex. STARD3 and STARD11 both contain a FFAT (two phenylalanines in an acidic tract) motif between their N-terminal membrane targeting determinants and the C-terminal START domain (Figure 2). Almost all START proteins have been implicated in either in cancer progression or suppression (Olayioye et al., 2004, 2005; Durkin et al., 2007a, b; Clark, 2012; Vassilev et al., 2015). Here we focus on the FFAT motif-containing proteins, STARD3 and STARD 11, and discuss their role in cancer.

### STARD3

STARD3 was originally named metastatic lymph node clone 64 protein (MLN64) since it was discovered in a screen designed to identify human genes that were amplified or overexpressed in aggressive breast tumor. The screen used subtractive hybridization method and identified clone number 64 as a gene that is overexpressed in all HER2 positive breast tumors (Tomasetto et al., 1995). Subsequently, it was shown to be co-amplified and co-expressed with HER2 in various breast cancer cell lines and in about 10–25% breast cancers (Bièche et al., 1996; Vassilev et al., 2015). STARD3 gene is located in the minimal amplicon of HER2-positive breast cancers. It is co-amplified with HER2 (Alpy et al., 2003) and always overexpressed with HER2 in breast cancer cells (Pollack et al., 1999; Perou et al., 2000; Vincent-Salomon et al., 2008).

**FIGURE 3:**
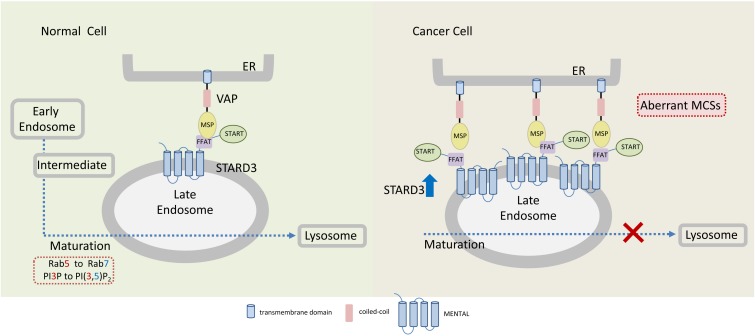
Endoplasmic reticulum-endosome MCSs in normal and cancer cells overexpressing STARD3. The sterol-transfer protein STARD3 promotes the formation of MCSs between late endosomes (LE) and the endoplasmic reticulum (ER), where it mediates cholesterol transport. Tethering of ER and LE occurs through the interaction of the LE-membrane anchored STARD3 (via its FFAT-like motif) with the integral ER proteins VAPs. In cancer cells, overexpression of STARD3 possibly induces the formation of aberrant LE-ER MCSs thereby inhibiting further endosomal maturation. Endosomal maturation is commonly associated with Rab5 to Rab7 switch and with PI3P to PI(3,5)P_2_. MSP, major sperm protein domain.

Currently, it is unclear how STARD3 enhances tumorigenesis of HER2-positive breast cancer and how the two proteins cooperate. However, several possibilities could be postulated; STARD3, via its cholesterol transfer activity, plays a central role in redistribution of cholesterol between the ER and endosomes. It interacts with the ER via its FFAT motif and with endosome via its MENTAL domain (Figure 3). The MENTAL domain shares structural homology with tetraspanin superfamily consisting of four transmembrane helices. This domain does not have any typical late endosome (LE)-targeting motifs, however, mutagenesis analysis strongly suggests that the MENTAL domain is crucial for STARD3 targeting to LE (Alpy et al., 2013). When STARD3 is amplified or overexpressed in HER2-positive breast cancer, the endosomal membranes are wrapped by the ER, leading to rigid and static ER-LE MCSs, thus losing their transient and dynamic features. Interestingly, stacking of ER membranes is also observed by ectopic overexpression of LTP proteins containing FFAT motif together with vesicle-associated membrane protein-associated proteins (VAPs) which produces abnormal ER structures called karmellae (Amarilio et al., 2004). The ER-LE static structures might lock the LE and inhibit their maturation to lysosomes (Figure 3). Under these conditions, lysosomal degradation of cell surface receptors, including HER2 and other growth factors receptors would be impaired, receptors will be sorted back to the PM and signal termination will be prevented, leading to uncontrolled cell growth. In this way, STARD3 may enhance the progression of HER2-positive cancer. Indeed, it was shown that STARD3 overexpression increases the proliferation rates of HER2-positive breast cancer cells, while its knockdown has an opposite effect (Wilhelm et al., 2017).

### CERT (STARD11)

**FIGURE 4:**
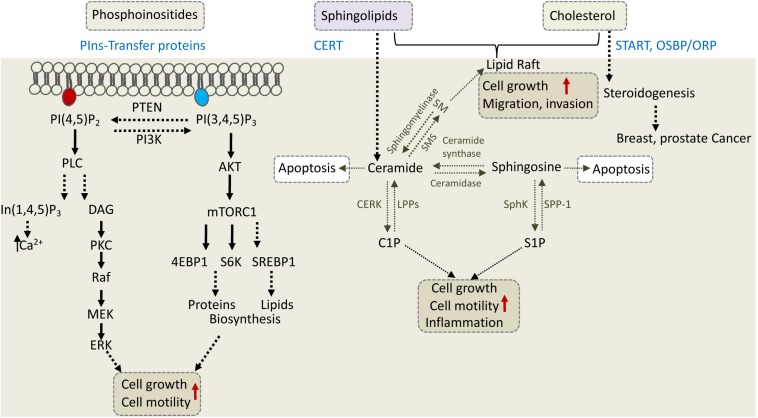
Phosphoinositides, sphingolipids, and cholesterol regulate cell growth, motility, and invasion. The depicted cellular pathways are regulated by phosphoinositeds (PIns), sphingolipids, and cholesterol and can influence cell growth, motility, invasion, or apoptotic cell death. LTPs are labeled in blue and include PIns-transfer proteins, ceramide transfer protein (CERT), and various cholesterol transfer proteins of the START and OSBP/ORP family. PLC, phospholipase C; PKC, protein kinase C; DAG, diacylglycerol; S6K, S6 kinase; SM, sphingomyelin; SMS, SM synthase; S1P, sphingosine 1-phosphate; C1P, ceramide 1-phosphate; LPP, lipid phosphate phosphatase; SPP-1, S1P phosphatase-1; CERK, ceramide kinase; SphK, sphingosine kinase.

CERT, a 68-kDa cytosolic protein, also known as collagen type IV alpha-3-binding protein (Col4A3BP) or STARD11, transfers ceramide from the ER to the Golgi, where various modifications take place to produce complex sphingolipids (Hanada et al., 2003). CERT via its N-terminal PH domain binds PI4P at the Golgi and via its FFAT motif interacts with the ER-resident VAP proteins to transfer ceramide through the ER-Golgi MCSs (Kawano et al., 2006; Peretti et al., 2008). The START domain of CERT is exclusively specific for ceramide. The significance of CERT in cell physiology and cancer progression is mainly associated with its ceramide transfer activity, as ceramide is a precursor of sphingolipids (Figure 4).

Sphingolipids are made up of a large class of lipid species having sphingosine as their backbone. They are involved in maintaining the structural integrity and fluidity of cell membranes and in regulating various cellular processes such as proliferation, migration, angiogenesis and inflammation (Kunkel et al., 2013; Morad and Cabot, 2013; Kreitzburg et al., 2018). Ceramide, an *N*-acylated form of sphingosine, is the simplest type of sphingolipid; it serves as a precursor of more complex sphingolipids, including sphingomyelin (SM), glycosylceramide and ceramide 1-phosphate (C1P), which are produced at the Golgi by SMS (sphingomyelin synthase), UGCG (UDP-glucose ceramide glucosyltransferase) and CERK (ceramide kinase), respectively (Yamaji and Hanada, 2015).

Sphingomyelin, which is synthesized by SMS from PC and ceramide, is a key component of lipid rafts, affects membrane fluidity and is involved in signal transduction. Of note, CERT was first isolated as a factor that recovers SM levels in a SM-deficient cell line (Hanada et al., 2003). Glycosylceramide is synthesized by UGCG via transferring a glucose residue from UDP-glucose to ceramide. It serves as a precursor for lactosylceramide, which is the precursor of most of glycosphingolipids except galactosylceramide and its derivates. C1P is a phosphorylated form of ceramide and it functions as an adaptor for type IVA cytosolic phospholipase A2 (cPLA2) to produce pro-inflammatory eicosanoids. Among the three sphingolipids, SM is mostly affected by CERT defects, although the other two are also influenced (Prestwich et al., 2008; Yamaji and Hanada, 2014, 2015).

The central role of ceramide in sphingolipid metabolism is also demonstrated in sphingosine-1-phosphate (S1P) pathway, which regulates multiple cellular processes such as proliferation, neovascularization, migration, and invasion. Ceramide, sphingosine and S1P comprise the three core lipids of S1P pathway, which are rapidly interconverted in response to various external stimuli such as growth factors, inflammation and stress. Ceramidase converts ceramide to sphingosine, which is further modified by SphK (sphingosine kinase) to S1P or reversed to ceramide by ceramide synthase (Figure 4). ABC transporters and Spns2 (spinster homolog 2) can export S1P outside the cell, where it binds to S1PR1 to 5 (sphingosine-1-phosphate receptor), and induces their signal transduction in both autocrine and paracrine manner (Spiegel and Milstien, 2003).

While ceramide induces apoptosis, its metabolites induce signaling cascades that promote cell proliferation or migration (Figure 4). Therefore, CERT can either promote or inhibit cancer progression depending on cellular context. In triple-negative breast cancer (TNBC), for example, CERT depletion promotes cancer progression (Heering et al., 2012). It was proposed that low levels of CERT in TNBC concomitant with reduced levels of SM and cholesterol at the PM, increased PM fluidity and caused high activation of EGFR (epidermal growth factor receptor) to enhance tumorigenesis (Heering et al., 2012). On the other hand, CERT depletion was beneficial for cancer therapy in colorectal and HER2-positive breast cancer cell line (Lee et al., 2012). CERT is highly expressed in HER2-positive breast cancer, and its depletion induced ceramide accumulation in the ER and concomitant changes in genes expression. One of the genes induced by CERT depletion was LAMP2 (lysosomal associated membrane protein-2) which mediated paclitaxel sensitization via induction of autophagic cell death (Lee et al., 2012). It appears that inhibition of CERT could lead to tumor suppression in some cancers and tumor progression in others, and thus could represent a potential target for precision medicine. Similar to CERT, other LTPs that regulate phosphoinositides, shingolipids and cholesterol can affect different signaling and metabolic pathways to enhance cell survival, growth and motility or to inhibit cell death, and consequently could affect cancer progression, metastasis and/or response to treatment (Figure 4).

## Membrane Contact Sites

MCSs are defined as small cytosolic gaps of ∼10–25 nm between the ER membranes and PM [plasma membrane-associated membranes (PAM)], the mitochondria [mitochondria-associated ER membranes (MAM)], or other intracellular organelles including endosomes, Golgi complex, peroxisomes, lysosomes and lipid droplets (Levine, 2004; Wong et al., 2018). These contact sites enable the transport of lipids, calcium ions and different metabolites by non-vesicular transport mechanisms, and thus, provide a platform for inter-organellar communication (Holthuis and Levine, 2005). MCSs are highly dynamic and heterogenous structures formed by specialized tethering proteins that bridge two membrane compartments (Lev, 2010). Multiple organelle-specific tethering complexes have been isolated (Scorrano et al., 2019) and many of them contain the integral ER-membrane proteins, VAP-A and -B (Lev et al., 2008).

VAP proteins interact via their major sperm protein (MSP) domain with FFAT motif-containing proteins, including the LTPs CERT, OSBP (oxysterol-binding protein 1) and Nir2 (Hanada, 2006; Peretti et al., 2008), and play major roles in MCSs formation between the ER and other cellular membranes (Murphy and Levine, 2016). Nevertheless, VAPs depletion has no profound effects on cell viability and contacts between ER and other organelles (Stoica et al., 2014; Dong et al., 2016), implying that other proteins are involved. Indeed, many tether proteins have been identified in the last few years, including the ER-anchored protein MOSPD2 (motile sperm domain-containing protein 2), which also interacts with FFAT-containing proteins and is implicated in MCSs formation (Di Mattia et al., 2018). Notably, MOSPD2 and VAP proteins have been shown to interact and possibly form hetero-oligomers (Huttlin et al., 2017).

The molecular components of the different MCSs, their function in communication and metabolic exchanges, make MCSs a subject of great interest in cellular signaling and metabolism in both physiological conditions and pathological contexts, such as cancer and neurodegeneration. Here, we address the features of specific types of MCSs (involving mitochondria, endosomes, and lysosomes) with a focus on their role as key platforms for calcium signaling and lipid transfer, especially in cancer.

## Mitochondria-Associated ER Membranes (MAM) and Its Role in Cancer

Mitochondria-associated ER membranes (MAM) specific MCSs that create an intimate communication between ER and mitochondria and generate micro-domains in which the concentration of Ca^2+^ is much higher than the cytosol (Csordás et al., 2010), allowing for rapid mitochondrial Ca^2+^ uptake through the low affinity (KD of 20–30 μM) channel of the mitochondrial calcium uniporter (MCU) (Baughman et al., 2011; De Stefani et al., 2011). Calcium uptake through the MCU complex covers essential roles in regulating energy status, signaling events and survival (Mammucari et al., 2016; Penna et al., 2018).

In the mitochondrial matrix, Ca^2+^ controls the activity of the three dehydrogenases of the Krebs cycle and, thus, the overall synthesis of ATP. Cancer cells, which require high energy for growth, commonly turn their energy production from oxidative phosphorylation to glycolysis (Warburg effect) (Schwartz et al., 2017). Although the amount of ATP produced via glycolysis is lower than through oxidative phosphorylation, it provides a selective advantage to cancer cells due to significantly higher glycolytic rate, supporting tumor growth and progression. Such a metabolic switch from aerobic metabolism to glycolysis has been linked to alterations of Ca^2+^ signaling at the MAM (Bittremieux et al., 2016). Dysregulation of calcium import at MAM can therefore severely affect tumorigenesis through two critical mechanisms: cellular metabolism and cell death pathways (Figure 3).

The current concept is that Ca^2+^ overload in the mitochondria leads to apoptosis, whereas basal level of Ca^2+^ enhances tumorigenesis. Indeed, several compounds with anti-tumor activity act by promoting mitochondrial calcium overload and consequently cell death, which can be inhibited by MCU blockers (Garcia-Prieto et al., 2013; Madreiter-Sokolowski et al., 2016). Likewise, inhibition of mitochondrial Ca^2+^ uptake enhances resistance to apoptotic stimuli in colon, cervical and prostate cancers, and increases cancer cell survival (Cui et al., 2019). However, in MDA-MB-231 breast carcinoma, MCU downregulation reduced tumor growth and metastasis, implying that mitochondrial Ca^2+^ uptake enhanced tumorigenesis of some cancers (Tosatto et al., 2016).

**FIGURE 5:**
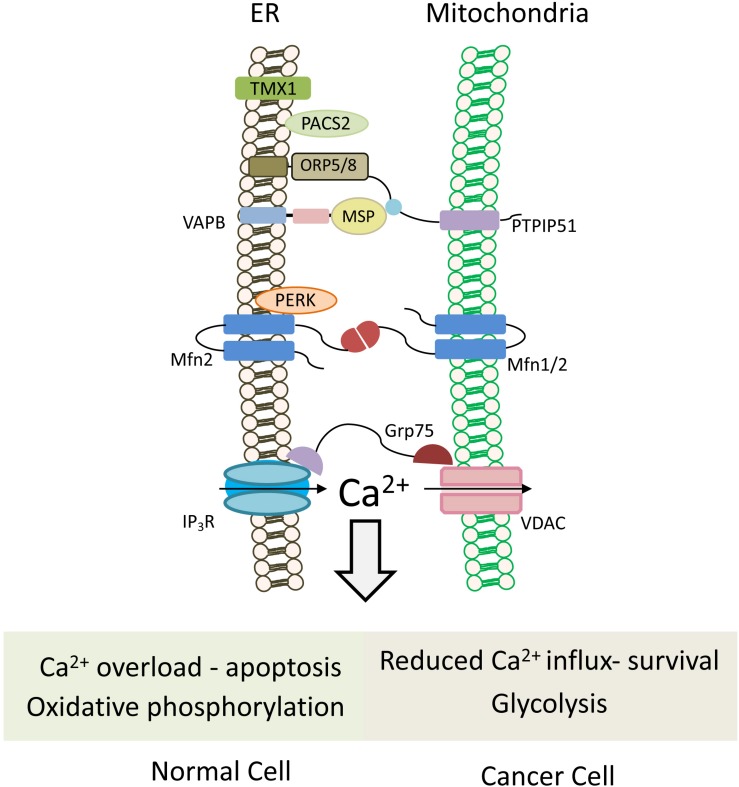
Mitochondria-associated ER membranes in normal versus cancer cells. Schematic cartoon illustrating ER-mitochondria (MAMs) tethering proteins. MAMs regulate lipid transfer and play an important role in Ca^2+^ homeostasis by orchestrating Ca^2+^ shuttling from ER to mitochondria. Normal cells rely on oxidative phosphorylation for energy production, and possess normal MAM configuration, which promotes apoptotic cell death in response to calcium overloading. Conversely in cancer cells, which use the glycolytic pathway to produce ATP, expression level of tethering proteins is altered and “aberrant” MAMs are formed. In most cases, the ER-mitochondria contact is reduced and, hence, also the mitochondrial calcium uptake, favoring cell survival and resistance to chemotherapeutic drugs. Multiple proteins are involved in ER-mitochondria tethering (Sassano et al., 2017), those that are described in the text and the figures are: TMX1, thioredoxin related transmembrane protein 1; PTPIP51, protein tyrosine phosphatase-interacting protein 51; VAPB, VAMP-associated protein B; Mfn1/2, Mitofusin 1/2; PERK, protein kinase RNA-like ER kinase; GRP75, glucose-regulated protein 75; IP_3_R, IP3 (inositol 1,4,5-trisphosphate) receptor; VDAC, voltage-dependent anion channel; PACS2, phosphofurin acidic cluster sorting protein 2.

Calcium is released from the ER through the IP_3_R, which is tethered to the mitochondrial VDAC1 via the GRP75 linker (Szabadkai et al., 2006; Figure 5). Several oncogenes modulate IP_3_R activity by post-translational modification or direct interaction. Phosphorylation of IP_3_R by AKT inhibits Ca^2+^ release and protects cancer cells from apoptosis (Szado et al., 2007). Similarly, interaction with the anti-apoptotic proteins Bcl-2 and Bcl-XL, which are frequently overexpressed in cancers (Delbridge et al., 2016), suppresses ER Ca^2+^ release to prevent apoptosis (Huang et al., 2013; Monaco et al., 2015; Morciano et al., 2018).

Different tether proteins have been postulated for MAMs formation and maintenance (Figure 5). Homo- and heterotypic interaction of Mitofusin 1 (MFN1) and 2 (MFN2) was initially proposed as a tether for MAM (Figure 5; De Brito and Scorrano, 2008). Despite both Mitofusins are transmembrane GTPases involved in mitochondrial fusion, MFN1 is localized to the outer mitochondrial membrane, while MFN2 is found both in the ER and mitochondria, largely present at MAM (De Brito and Scorrano, 2008; Naon et al., 2016). High MFN2 level in cancer cells was proposed to increase MAM and enhance ER-mitochondria Ca^2+^ flux and hence, susceptibility to apoptosis (Gautier et al., 2016; Cui et al., 2019). Interestingly, MFN2 also physically interacts with PERK (protein kinase RNA-Like ER kinase) (Muñoz et al., 2013), which also functions as a tether at MAM extensions (Verfaillie et al., 2012). In cancer cells, PERK may promote or suppress tumor progression. In the mesenchymal subtype of TNBC, PERK signaling enhanced invasion and metastasis through interaction with the transcription factor CREB3L1 (cAMP responsive element binding protein 3 like 1) (Feng et al., 2017), and its knockdown inhibited growth of breast carcinoma in animal models by limiting redox homeostasis (Bobrovnikova-Marjon et al., 2010).

Phosphofurin acidic cluster sorting protein 2 (PACS-2) is a sorting protein that also functions as a MAM tether, and is involved in ER-mitochondria coupling (Simmen et al., 2005), as well as in apoptosis and survival. Apoptotic signals trigger its dephosphorylation and redistribution from the ER to mitochondria, recruiting Bid, followed by Bid cleavage and cell death (Simmen et al., 2005), while its phosphorylation by AKT promotes NF-kB (nuclear factor kappa-light-chain-enhancer of activated B cells)-mediated pro-survival signaling (Betz et al., 2013).

Among the MAM proteins that regulate ER-mitochondria Ca^2+^ flux and affect cancer cells, are the redox-sensitive oxidoreductase thioredoxin related transmembrane protein 1 (TMX1) and protein tyrosine phosphatase-interacting protein 51 (PTPIP51). Reduced levels of TMX1 in cancer cells lead to increased ER Ca^2+^ levels, and a concomitant decrease in cytosolic and mitochondrial Ca^2+^ levels resulting in reduced mitochondrial respiration. This, in turn, makes the cancer cells more dependent on glycolysis, a hallmark of cancer cells (Ganapathy-kanniappan and Geschwind, 2013).

PTPIP51, an integral outer mitochondrial membrane (OMM) protein, interacts with VAP-B and is essential for VAP recruitment to MAM (Figure 5). It also interacts with the oxysterol-binding protein (OSBP)-related proteins ORP5 and ORP8, which transfer phosphatidylserine (PS) to the mitochondria for PE synthesis (Galmes et al., 2016). Depletion of PTPIP51 or VAP-B delays Ca^2+^ uptake by the mitochondria (De vos et al., 2012). Notably, both PTPIP51 and VAP have growth stimulatory activities, and high expression level of VAP-B in breast cancer enhanced cell growth *in vitro* and tumor growth in animal models (Rao et al., 2012).

Collectively, these examples demonstrate that many MAM proteins can influence tumor metabolism and/or apoptotic cell death and consequently may affect tumorigenesis or response to anti-cancer therapy.

### Lipids Modifications at the MAMs and Their Role in Cancer

The role of MAM in the synthesis of specific lipids and their transfer to mitochondria was initially shown via cell fractionation (Vance, 1990; Vance and Canada, 1991). MAM is essential for the conversion of ER-derived PS to PE and for trafficking of cholesterol as a precursor for steroid species (Tatsuta et al., 2014).

Although mitochondria have low content of cholesterol compared to other organelles, cholesterol is enriched in MAMs compared to the rest of the ER and affects ER-mitochondria apposition (Sassano et al., 2017). In cancer cells, the inner mitochondrial membrane (IMM) has higher cholesterol content and phospholipids with shorter and more saturated acyl chains compared to normal cells. These lipid modifications decrease the IMM permeability, and consequently the vulnerability to apoptotic signals (Ribas et al., 2016).

Cardiolipin is a unique and abundant lipid of the IMM, accounts for ∼20% of the total lipid composition, which retains cytochrome *c* in the IMM (Shidoji et al., 1999). Its accumulation in the IMM requires PA supply mediated by the PA-transfer activity of the TRIAP1/PRELI protein complexes. Depletion or inhibition of these protein complexes impairs cardiolipin accumulation and increases cell susceptibility to apoptosis (Potting et al., 2013). Hence, it could be that “aberrant” MAMs in cancer cells or abnormal expression of TRIAP1/PRELI would modulate cardiolipin levels and cytochrome *c* release, and thus cell susceptibility to apoptosis that can be exploited for cancer therapy.

## Role of ER-Endosome and ER-Lysosome Contact Sites in Human Cancer

The endosomes undergo dynamic changes from biogenesis toward maturation. Endosome maturation is mediated by spatiotemporal phases, which regulate their size, location, uptake of macromolecules and sorting of cargos. The number of ER-endosomes MCSs is markedly increased during maturation, reaching a maximum in the LE (Friedman et al., 2013; Hariri et al., 2016). We describe the functions of key proteins that are involved in ER-endosomes MCSs and their putative implications in cancer.

In addition to STARD3, the retromer subunit SNX2 (sorting nexin-2) also interacts with VAPs and tethers the ER membrane to endosomes (Dong et al., 2016). SNX2 binds PI(3)P on the endosomal surface, and affects the level of several cell surface proteins in cancer cells, including the c-Met receptor in lung and gastric cancer cells (Ogi et al., 2013). Depletion of VAPs leads to accumulation of PI4P in endosomes and disrupts endosome-to-Golgi traffic. VAPs also interact with the ER proteins Protrudin and RTN3 (Reticulon protein 3), while Protrudin interacts with RAB11 (recycling endosomes), Rab7 (late endosomes) and PI(3)P at the endosomes via its FYVE domain (Shirane and Nakayama, 2006; Matsuzaki et al., 2011). Overexpression of Protrudin increases ER-endosomes contacts (Raiborg et al., 2015), while resistance to endocrine therapies of breast cancer cells is associated with reduced levels of Protrudin (Magnani et al., 2013). Rab7 was also shown to be a marker of poor prognosis in melanoma cancer (Alonso-Curbelo et al., 2014). Whether Protrudin overexpression in cancer induces aberrant MCSs is currently unknown, but could be interesting to explore.

Another protein that functions at the ER-endosome MCSs is the ER-localized protein tyrosine phosphatase PTP1B which interacts with EGFR on early and late endosomes at the ER-endosome MCSs (Eden et al., 2010). EGFR is implicated in various human cancers, while PTP1B can function either as an oncogene or tumor suppressor in various cancer types (Liu et al., 2015). At the ER-endosomes MCSs, PTP1B-EGFR interaction stabilizes MCSs, but it is not required for contact formation (Eden et al., 2010). As EGFR is highly expressed in many human cancers, it might stabilize aberrant ER-endosome MCSs to sustain endosomal signaling and prevent signaling termination by lysosomal degradation.

The lysosomes participate in many fundamental cellular processes, including recycling of cellular components, nutrient-dependent signal transduction, membrane repair and pathogen defense signaling (Perera and Zoncu, 2016). Increased lysosomal activity, especially under nutrient deprivation, favors cancer growth and resistance to therapy in certain cancer types (Thelen and Zoncu, 2017). Lysosomes are considered as a central hub for sorting of lipids from endogenous and exogenous origin, and for maintenance of cholesterol homeostasis (Thelen and Zoncu, 2017). Another important property of the lysosomes is the close proximity of 5–20 nm with other organelles including the ER and mitochondria (Csordás et al., 2006; Phillips and Voeltz, 2016; Wong et al., 2018).

Lysosomes can process and distribute exogenous (LDL-cholesterol) and endogenous (*de novo* synthesized in the ER) cholesterol through MCSs. The ER-anchored protein ORP5 and the membrane cholesterol transporter NPC1 (Niemann-Pick disease, type C1) interact and facilitate cholesterol export from lysosomes, whereas STARD3 in the LEs/Lys, through interactions with VAPs, mediates cholesterol transport from the ER to lysosomes (Thelen and Zoncu, 2017). ORP5 promotes cell proliferation and invasion via mTOR complex 1 (mTORC1) signaling (Du et al., 2018), and its overexpression is associated with poor prognosis of pancreatic cancer (Koga et al., 2008). Interestingly, ORP5 and ORP8 were also localized to MAM (Gao and Yang, 2018), similar to the ER protein PDZD8 (PDZ domain-containing protein 8) (Hirabayashi et al., 2017), which was recently found at the ER-LEs/Lys contacts through interaction with Rab7 (Guillén-Samander et al., 2019). It was proposed to regulate Ca^2+^ dynamics in neurons and lipid transport between the ER and ER-LEs/Lys (Hirabayashi et al., 2017; Guillén-Samander et al., 2019).

In addition to cholesterol distribution, the ER-lysosome MCSs promote efficient Ca^2+^ transport between the two organelles. It is now clear that many functions of lysosomes depend on their ability to acquire calcium from the ER through IP_3_Rs and to release calcium (Atakpa et al., 2018). Lysosomal calcium release was proposed to be mediated by three types of channels: the mucolipin family of TRPML (transient receptor potential) channels, the two-pore (TPC) channels, and the transient receptor potential cation channels TRPVs (Raffaello et al., 2016; Li et al., 2019). Interestingly, TRPV4 is associated with poor prognosis in colon cancer (Liu et al., 2019) and is implicated in breast cancer metastasis (Lee et al., 2016). Similarly, TPCs have been found to be highly expressed in several cancers (Brailoiu et al., 2009; Jahidin et al., 2016) to facilitate cell migration and invasion (Nam et al., 2017).

ER-lysosome MCSs also play role in mTOR activation. mTOR is a central regulator of cell metabolism and growth, and is considered as a promising target for cancer therapy (Faes et al., 2017; Saxton and Sabatini, 2017). mTOR is activated at the LE/LY in response to multiple growth factors and amino-acid stimulation. Its activation is regulated by lysosomal positioning and is mediated by translocation of mTORC1-positive lysosomes to the cell periphery, where it remains in proximity of signaling receptors. It turns out that this translocation is regulated by ER-lysosome MCSs, and is mediated by two PI3-binding proteins: FYCO1 (FYVE and coiled-coil domain-containing protein 1) which is recruited to lysosomes, and the ER-resident protein Protrudin. PI3P-binding of FYCO1 and Protrudin promotes mTORC1 activation and concomitantly inhibits autophagy (Hong et al., 2017).

Overall, these findings suggest that ER-lysosome MCSs can affect fundamental properties of cancer cells including growth and metabolism, which may have aberrant configurations in cancer.

## Concluding Remarks

In contrast to normal cells, cancer cells are characterized by distinct cellular metabolism and uncontrolled cell growth, migration and invasion. Many of these processes are influenced by lipids and calcium, two critical second messengers, which are regulated by LTPs and MCSs. LTPs can modulate the levels of lipid second messengers and thus can modify signaling pathways, signaling duration and termination. LTPs can also modulate the distributions of lipids, and consequently the stiffness, fluidity, and permeability of membranes, therefore affecting cell adhesion, receptor endocytosis and recycling, cell growth and migration as well as susceptibility to cancer therapy. Identification of specific LTPs that regulate these cellular processes which are aberrantly expressed in human cancer could be used for therapeutic intervention. Similarly, MCSs which affect lipid and calcium homeostasis, have an impact on cell proliferation and growth. On the other hand, calcium and certain lipids are involved in stress response and cell death pathways. The challenge is to switch off abnormal function or expression of LTPs in cancer cells and/or to direct “aberrant” MCSs toward cell death rather than cell proliferation, by manipulating the different tethering mechanisms that regulate MCSs formation and stability. Further studies on MCSs configuration and LTPs functions in cancer cells will be able to shed more light on how they may affect cell transformation and promote cancer development and metastasis.

## Author Contributions

DP wrote the text related to MCSs sections and incorporated the references. RT wrote the text on MCSs, mitochondrial function and related figure legends, and edited the manuscript. SK wrote on START family and prepared the related figure. SL was responsible for the other text sections, figures, and integrating the review. All authors listed have made a substantial, direct and intellectual contribution to the work, and approved it for publication.

## Conflict of Interest

SK was employed by company Nakseongdae R&D Center. The remaining authors declare that the research was conducted in theabsence of any commercial or financial relationships that could be construed asa potential conflict of interest.
